# The Prosodic Characteristics of Non-referential Co-speech Gestures in a Sample of Academic-Lecture-Style Speech

**DOI:** 10.3389/fpsyg.2018.01514

**Published:** 2018-09-07

**Authors:** Stefanie Shattuck-Hufnagel, Ada Ren

**Affiliations:** Speech Communication Group, Research Laboratory of Electronics, Massachusetts Institute of Technology, Cambridge, MA, United States

**Keywords:** co-speech gesture, speech prosody, speech production planning, prosodic prominence, prosodic constituents

## Abstract

Many studies have documented a close timing relationship between speech prosody and co-speech gesture, but some studies have not, and it is unclear whether these differences in speech-gesture alignment are due to different speaking tasks, different target gesture types, different prosodic elements, different definitions of alignment, or even different languages/speakers. This study contributes to the ongoing effort to elucidate the precise nature of the gesture–speech timing relationship by examining an understudied variety of American English, i.e., academic-lecture-style speech, with a focus on an understudied type of gesture: Non-Referential gestures, which make up the majority of this corpus. Results for the 1,334 Stroke-Defined Gestures in this 20-min sample suggest that the stroke phase of a Non-Referential gesture tends to align with a pitch-accented syllable, just as reported in studies of other gesture types (e.g., deictic gestures) and in other speaking styles (such as narration). Preliminary results are presented suggesting that trajectory shapes of these Non-Referential gestures are consistent across a higher-level prosodic grouping, supporting earlier proposals for kinematic constancy across spoken prosodic constituents (Kendon, 1972, 1980, 2004). Analysis also raises the possibility that the category of Non-Referential gestures is not solely made up of ‘beats,’ defined as simple bi-phasic flick-like movements that beat out the rhythm of the speech, but includes gestures with multiple phases and various types of rhythmicity. Taken together, the results of this analysis suggest (1) a wide range of gesture configurations within the undifferentiated category of Non-Referential gestures or ‘beats,’ which requires further investigation, and (2) a close coordination between co-speech gestures and the prosodic structure of spoken utterances across speaking styles and gesture referentiality, which has profound implications for modeling the process of planning an utterance.

## Introduction

The relationship between spoken utterances and the co-speech gestures that often accompany them has been the subject of great interest over the centuries, and this interest has intensified with the development of modern prosodic theory. Over the past few decades, the incorporation of phrase-level prosodic constituency and prominence patterns into linguistic grammars (e.g., Liberman and Prince, 1977; Selkirk, 1984; Beckman and Pierrehumbert, 1986; Nespor and Vogel, 1986), along with the development of an extensive system for capturing significant systematic aspects of gestural movements and their communicative function (Kendon, 1972, 1980, 2004; McNeill, 1992, 2005) has opened the door to a range of studies asking how these two streams of behavior interact. This is an important question, because to the extent that both sets of actions contribute to the communication of a message during the act of speaking, it is a reasonable presumption that they are planned together (Esteve-Gibert and Prieto, 2013; Krivokapic, 2014; Wagner et al., 2014; Krivokapic et al., 2015; Esteve-Gibert et al., 2017). Such a view has critical implications for the development of a comprehensive model of the speech production planning process. Moreover, evidence that the two sets of actions are closely timed with respect to each other has the potential to implicate a prosodic representation as the integrating planning framework for both speech articulation and co-speech gesture, since it is increasingly apparent that prosodic structure is one of the major factors governing speech timing (Wightman et al., 1992; Byrd et al., 2006; Turk and Shattuck-Hufnagel, 2007 inter alia).

In his influential 1992 book *Hand and Mind*, David McNeill proposed a categorization scheme for co-speech gestures that separated Referential gestures, which visually illustrate some aspect of the semantic content of the speech they accompany, from Non-Referential gestures, which can be said to convey information about the form of the utterance rather than its content. Referential gestures have been subdivided into various types (McNeill, 1992), including iconic (illustrating concrete aspects of the speech content), metaphoric (illustrating abstract aspects), and deictic (pointing to actual or symbolic locations). These Referential gesture subclasses, based on the philosophical work of Pierce (1960; McNeill and Levy, 1982), have understandably been of particular interest, because their relationship to the meaning of the speech is often straightforward to identify. Moreover, this relationship is compelling as an argument for the integration of the planning for speech and gestural movements as co-signaling systems for the meaning of an intended message. Thus, Referential gestures have been extensively explored in a wide range of studies, which have revealed their striking contribution to acts of communication. In contrast, Non-Referential gestures, which are often called ‘beats’ (or sometimes, ‘batons,’ Efron, 1941/1972), have not been as extensively subcategorized. The term ‘beats’ suggests a degree of rhythmic periodicity, invoking a conductor beating out the rhythm of an orchestral performance, and Non-Referential gestures have sometimes been defined in these terms, as e.g., beating out the rhythm of the speech. Alternatively, McNeill (1992) describes a particular kind of beat, i.e., a single in-out or up-down flick of the finger or hand, which he notes can mark particular locations in a narrative structure. But Non-Referential gestures or beats have been primarily defined as ‘not iconic, metaphoric or deictic,’ leaving a substantial gap in our understanding of the range of behaviors in this set of gestural movements.

Although timing with respect to spoken prosody has been particularly important for Non-Referential gestures or beats, because they have been defined in terms of their relationship to the rhythm of speech, i.e., to the pattern of prominences in an utterance, Referential gestures have also been described as temporally aligned with the prosodic structure of speech. For example, Kendon (1972, 1980) proposed a hierarchy of prosodic units, from tone groups to locutions, locution groups, locution clusters and the discourse, and a corresponding hierarchy of gestural structures, from gesture phrases to gestural units. In a short sample of videoed conversation that he analyzed in great detail, he reported that these two sets of units were closely coordinated, such that, e.g., gesticular movements in successive tone groups differ in some characteristics, while sharing other characteristics if they formed a larger constituent, a locution (generally a full sentence). He noted that co-speech gestures may illustrate objects or actions referred to in the speech, or they may indicate the organizational structure of the elements of the discourse. Thus he did not distinguish sharply between Referential gestures that visually illustrate an aspect of the speech, and Non-Referential gestures that have other functions, in their likelihood of aligning with prosodic structure.

Other investigators who have focused on gesture-prosody alignment have also looked at co-speech gestures as a single category, without distinguishing between Referential and Non-Referential categories. For example, Loehr (2004) reports temporal alignment between gestural strokes and spoken pitch accents (i.e., phrase-level prominences signaled by F0), without distinguishing among gesture types, and Shattuck-Hufnagel et al. (2007) report similar findings for gestures with sudden sharp end points (which they called ‘hits’). Investigations have sometimes focussed on the alignment of particular subtypes of Referential gestures, particularly deictic or pointing movements, and eyebrow movements (Krahmer et al., 2002; Keating et al., 2003), that appear to have a prominence-lending function. Thus the question of how Non-Referential gestures, as a specific subset of co-speech gestures, align with spoken prosody has not been thoroughly investigated. This paper reports some preliminary results from a larger study aimed at extending our current understanding of the relationship between the prosody of a spoken utterance and Non-Referential co-speech gestures in an understudied speaking style, i.e., formal academic lectures. Initial informal observation suggested that this style elicits a large proportion of Non-Referential gestures, which also provides an opportunity to begin to examine the range and structure of this category of gestures, which appears not to be homogeneous. Thus the research questions addressed in this paper are (1) Are Non-Referential gestures the predominant type in this speech sample? (2) Do the Non-Referential gestures exhibit alignment with prosodic structure? And (3) Are Non-Referential gestures a homogeneous category, as suggested by their designation as ‘beats’?

## Materials and Methods

In the course of designing and carrying out this study, two issues have come to the fore. The first concerns the question of how to convey, in visual terms, the path of a gestural movement. Many different conventions have been used in the literature to capture on the printed page the dynamic aspects of a movement, which the viewer can easily discern when watching the speaker in person or watching a video. But none of these existing conventions seemed precisely satisfactory for our purposes. To supplement these conventions, we have developed a tool called the gestural sketch, which is a simple line drawing of the path that the hand traverses during a continuous sequence of gestures. This tool plays an important role in describing the degree of similarity or dissimilarity between successive gestures, as well as the trajectory shapes for gesture sequences that are perceived as beatlike.

The second issue concerns the size of the prosodic constituent in the speech that is most useful for reporting our results on gesture grouping. The prosodic hierarchy for spoken utterances is generally taken to have the Utterance as its highest constituent. Each Utterance is made up of one or more Full Intonational Phrases (marked by a Boundary Tone on the final syllable), with each Full Intonational Phrase made up of one or more Intermediate Intonational Phrases (marked by at least one Pitch Accent (phrase-level prominence) and a Phrase Tone controlling the fundamental frequency contour between the final pitch accent and the end of the phrase), and so on down the hierarchy (see Shattuck-Hufnagel and Turk, 1996 for a summary). On this view there are clear definitional characteristics that permit the identification of Full and Intermediate Intonational Phrases in the signal, but it is less clear what marks the edges of an Utterance or of even higher-level constituents in the hierarchy, such as the Locution or the Discourse (see Kendon, 1980 for discussion). This problem was addressed here by extending an existing method for prosodic annotation called Rapid Prosodic Transcription (RPT), which was developed by Cole et al. (2010). Extending the RPT method produced a ‘crowd-sourced’ identification of the higher-level constituents that were required for our study.

Although studies of the temporal alignment of co-speech gestures have generally found a close relationship between gesture timing and prosodic timing, this is not always the case (McClave, 1994; Ferre, 2005, 2010). This raises the question of whether different types of gestures and/or different types of speaking show different patterns of alignment. However, it is difficult to address this question because different studies have looked at different speaking tasks (e.g., spontaneous conversational speech, emotional speech, speech elicited in the laboratory via highly constrained tasks) and different types of gestures (often deictic), as well as different parts of a gesture [e.g., the gesture stroke defined as the high-intensity movement to a target (Yasinnik et al., 2004)] vs. the gesture stroke as the period during which the arm maintains it maximum extension in a deictic gesture, vs. the apex (Jannedy and Mendoza-Denton, 2005) and different locations in the spoken prosody (e.g., the discrete point of maximum F0 for a high pitch accent, vs. the time interval of the accented syllable). In addition, different speakers appear to produce different proportions of gesture types (Myers, 2012, described in Krivokapic, 2014), and findings appear to differ across languages. This rich variety in sample materials and methodological approaches and results has resulted in a range of findings (see Krivokapic, 2014; Wagner et al., 2014 for reviews) that suggest the need for a comprehensive comparison of timing patterns across speaking tasks, gesture types and prosodic structures, to determine the generalizability of individual findings. Such comprehensive coverage is a very long-term project; the study described in this paper contributes to this long-term goal by focussing on a sample of an understudied speaking style (academic lectures) in which the gestures are predominantly Non-Referential. The analysis includes alignment of the gestural strokes both with prosodic prominences and, in a preliminary way, with higher-level prosodic constituents. Results point the way to further studies to elucidate how speech and co-speech gestures interact in a communicative event, and they suggest some constraints on the set of appropriate models of the planning process that produces such an event.

The analyses carried out in this study required the hand-labeling of a wide range of characteristics of both the speech and the co-speech gestures. This labeling process provides the information that is necessary in order to test hypotheses about how these two streams of behavior are aligned with each other, and will be described in some detail.

### The Corpus

**FIGURE 1:**
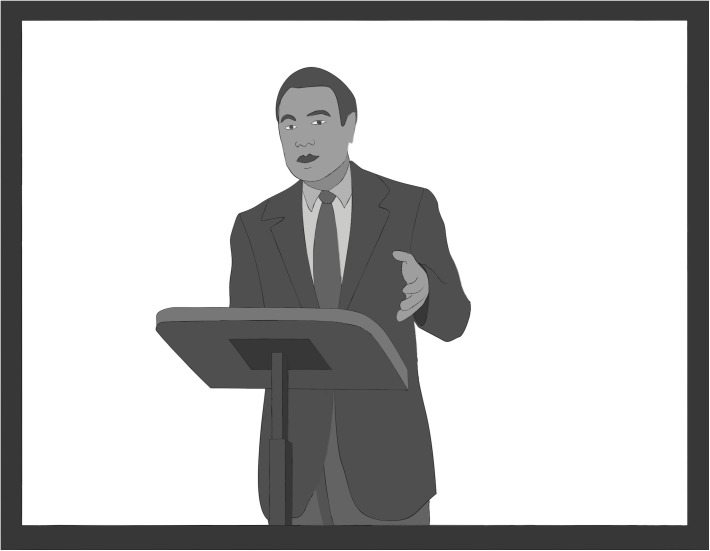
Illustration of the view of the speaker provided by the video samples analyzed in this study. For most of the sample, the speaker’s upper torso is visible, including the full extent of the arms and hands, enabling the annotation of the co-speech gestural movements in 2-dimensional space.

The availability of a video-recorded speech sample that provides a high proportion of Non-Referential gestures was discovered by accident, when a set of commercially available academic lectures (available from The Teaching Company/Great Courses Company^1^) was chosen as an object of study. According to information provided by the company, these lecturers are selected for their popularity on their respective campuses, and recruited to deliver a course in half-hour lectures to a small audience that is physically present in the room. The lectures are recorded on video and offered for sale to the public. It can be presumed that the lecturers selected in this way are effective communicators, and in our experience they generally produce fluent speech as well as large numbers of co-speech gestures. The sound quality of the recordings is also high, facilitating transcription of the utterances as well as annotation of their prosody. These videos were originally selected for the study of gesture-prominence alignment in part because they provide a clear view of the speaker’s upper body (**Figure 1**), which is filmed directly from the front. As a result, most of the time it is possible to view the full extent of the hands, arms, head and upper torso (at least when the speaker is not occluded by an illustrative graphic). In addition, these highly practiced college professors produce their lectures quite fluently, so that prosodic analysis of the prominences and word groupings in their speech is less challenging than for more typical speech, with its hesitations, restarts and other disfluencies. As we began to label the temporal locations of the gestures in these videos, we noticed that a large proportion of the gestures did not appear to be Referential, in the sense of visually illustrating the content of the accompanying speech in any obvious way. Thus was born the idea of analyzing this set of gestures with respect to its alignment with spoken prosody, in order to compare the results with existing observations of these alignment patterns for gestures which were either explicitly Referential or not distinguished with respect to their referential nature.

The subsample from the larger study that will be discussed in this paper includes an entire 30-min lecture produced by one male speaker (here referred to as the London sample, after its topic), which comprises 30 min 47 s of speech, with 23 min 35 s of video useable for gesture analysis. (For the excluded 7 min 12 s the speaker was not visible due to the display of illustrative graphics. The word transcriptions and prosody of the excluded portions of the sample are available for future analysis of the discourse structure of the lecture.)

### Labeling

In this paper, we focus on the manual co-speech gestures in the sample, i.e., those that involve the hand(s) and arm(s) of the speaker [The co-speech movements of other articulators, such as the head, eyebrows, direction of gaze and upper torso, are also of interest for their alignment with spoken prosody (e.g., McClave, 2000; Keating et al., 2003; Shattuck-Hufnagel et al., 2010; Swerts and Krahmer, 2010; Esteve-Gibert et al., 2017) but will not be discussed here]. For most aspects of the labeling, the gestures were annotated without listening to the speech, and the speech without viewing the video, to avoid any possibility of the labeler’s judgment about events in one channel being influenced by events in the other. However, this was not possible for one type of annotation, i.e., determining the referentiality of the co-speech gestures; this required the labeler to listen and look at the same time, because the decision depends on the relationship of the gestures to the meaning of the speech. Unless otherwise noted, each type of gestural annotation described below was carried out while viewing the silent video, and each type of speech annotation while listening to the sound recording only.

#### Gesture Annotation

The core of this study concerns the annotation of meaningful co-speech gestures, based on an exhaustive analysis of all movements made by the speaker during a lecture. Such annotation is not a trivial matter. Movements which occur during the speech, but can be regarded as not planned to be part of a communicative act, must be identified as such, and distinguished from intentional movements that appear to be part of a communicative act. These include movements such as grasping the podium, reaching out to turn a page, or very small ‘drifting’ movements of the hands or fingers, in which the articulator moves slowly in space in what appears to be a non-directed way. [There are gestures that can be interpreted as information by listeners, such as self-grooming actions like tucking the hair behind the ear or tossing the head, and may even in some cases be planned by the speaker to communicate information (such as flirtatiousness), but such movements are not included here. The question of whether even movements in this category are aligned with the spoken prosody is left for another day.] For the purposes of this study, we define movements planned to be part of a communicative act as those which include a stroke, i.e., an intentional movement that is sometimes referred to as ‘the business portion’ of a co-speech gesture. Thus the first annotation step was to identify the set of movements that each include a stroke, i.e., the set of Stroke-Defined Gestures (SDGs); this category defines the set of movements analyzed in this study. All of the gesture annotations were carried out by the second author, who is highly experienced in gesture labeling. Additional information on the suite of labeling methods can be found at http://adainspired.mit.edu/gesture-research/.

##### Gesture strokes

Gesture strokes were identified using the ELAN annotation software^2^. As noted above, strokes were distinguished from a number of other movement types, such as small undirected movements that lacked a sense of intentionality, task-related movements and drifting movements. The time of onset and offset of each stroke movement was annotated in a Stroke tier in ELAN.

Once the strokes are identified, specifying the Stroke-Defined Gestures (SDGs), a number of additional labeling steps can be carried out. Annotation results that will be reported here include the Referentiality of the gesture, its handedness, and for sequences of gestures, their perceived grouping. A number of additional characteristics have also been labeled for this sample, including, e.g., the optional gesture phases (i.e., preparation, pre-stroke hold, post-stroke hold and recovery, as proposed in Kendon, 1980, 2004); handshape (and change in handshape); trajectory shape (straight, curved or looping, i.e., forming a closed curve); and location with respect to the speaker’s body; these results will be reported in a subsequent publication and will not be discussed further here.

##### Gesture referentiality

Referentiality was labeled for each Stroke-Defined-Gesture, using an annotation scheme that included two categories: Referential and Non-Referential. As noted above, this labeling task (unlike the remaining tasks) was carried out while both viewing the video and listening to the speech.

##### Gesture handedness

Gesture handedness was labeled with an annotation scheme that included Left and Right for gestures made with one hand; two-handed gestures were labeled as Bimanual-synchronous or Bimanual-asynchronous (i.e., the two hands do not produce symmetrical movements), and Bimanual-L-dominant or Bimanual-R-dominant.

##### Perceived gesture groupings

As part of the larger ongoing study, sequences of gestures that were perceived as occurring in a group were labeled as Perceived Gesture Groups (PGGs), while looking at the silent video. This terminology was adopted instead of Kendon’s ‘Gesture Units,’ because Gesture Units are proposed to conclude with a relaxation phase, and it was not yet certain that the gesture sequences perceived as grouped had this characteristic. These PGGs formed the basis for analysis of the corresponding Gesture Sketches described below.

##### Gesture sketches

**FIGURE 2:**
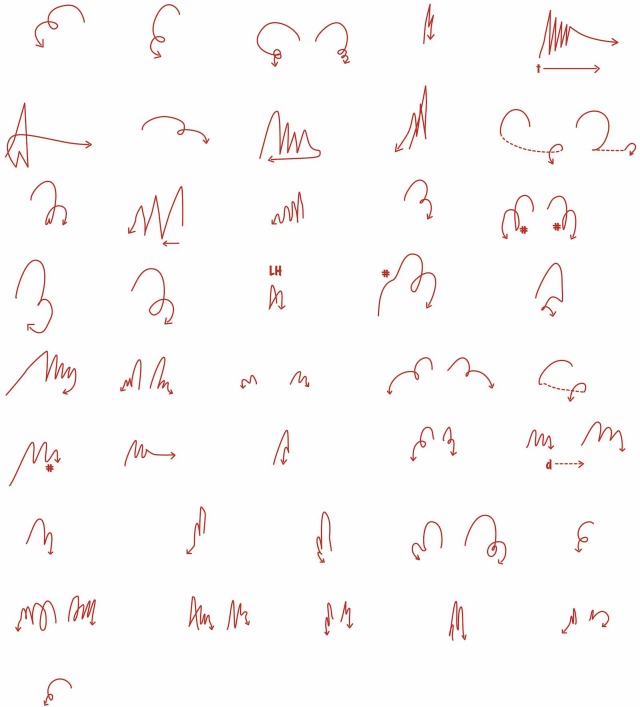
These gesture sketches of the first 43 sets of gestures in the London video sample show the paths of movement and handedness of individual groups of gestures defined by perceptual labeling; see below for discussion.

Gesture sketches were developed to provide a visual impression of the trajectory of a gestural movement, and to explore the possibility that they could facilitate judging the similarity in this characteristic across a sequence of gestures. Gesture sketches are line drawings of the trajectory through space of the moving hand, illustrated in **Figure 2** above. They provide a more detailed sense of the sometimes-complex path of movement of the hand through space than is possible using either a single-word characterization of path shape, or a short arrow added to a drawing of the speaker indicating direction of movement. They do not capture additional aspects of the movement, such as its timing, changes in velocity over time or its alignment with the speech, but for the purposes of this study they have proven to be a useful indicator.

Additional gesture characteristics and components that have been annotated, but are not discussed in this paper include gesture phases (Kendon, 1980, 2004), handshape, trajectory shape, and location with respect to the speaker’s body; these labels are designed to facilitate the quantitative estimation of similarity between one gesture and the next, and to test hypotheses about the cues to gesture grouping (Shattuck-Hufnagel and Ren, 2012). For some of the samples in the larger study, movements of the head, eyebrows and upper torso have also been annotated (Shattuck-Hufnagel et al., 2010), to facilitate comparison of the prosodic timing of gestures by various articulators, and investigation of the coordination among co-speech movements of various body parts.

#### Speech Annotation

To determine the timing relationship between the co-speech gestures and the prosodic constituent and prominence structure of the speech, the speech was transcribed orthographically and labeled for its intonational structure using Praat^3^ as a display and labeling tool and ToBI^4^ as the prosodic annotation system. This annotation was carried out by the first author, who is an experienced ToBI labeler. ToBI labels include, among other prosodic characteristics, the nature and location of tonal targets that signal phrase-level prosodic prominences (pitch accents) and two levels of intonational phrasing: higher-level Full Intonational Phrases, and the lower-level Intermediate Intonational Phrases that make up the higher level phrases.

In addition, to facilitate analysis of the temporal overlap between gestural strokes and pitch-accented syllables, a rough segmentation of the speech wave form into syllables was carried out. This segmentation task is challenging for utterances in English, where the syllable affiliation of an inter-vocalic consonant in words like *movie* or *label* is not always clear, but an approximate segmentation was carried out despite this difficulty.

Finally, when initial analyses made it clear that a larger prosodic constituent than the Full Intonational Phrase would be necessary in order to reach a clearer understanding of the relationship between the grouping of successive gestures and the prosodic constituent structure of the speech, a method for annotating the higher-level prosodic constituents was developed. This approach uses an extended version of the RPT, developed by Cole et al. (2010, 2014), in which untrained participants listen to recorded utterances and mark them (in real time) for prominence and word-group boundaries. In RPT, listeners mark only one size or level of boundary, and the annotations of multiple listeners are summed to provide a continuous-valued estimate of the size or level of the constituent boundary. While it is unclear exactly what criteria listeners use in determining the location of the boundaries they mark, since the full range of semantic and syntactic as well as prosodic information is available to them, Cole et al. (2010, 2014) have reported good agreement among listeners and a correlation of Rapid-Prosodic-Transcription-defined groupings with intonational phrases and prominences annotated by highly trained ToBI labelers. We extended this method by inviting listeners to mark three levels of constituent boundary rather than just one level, using a single slash (/) for the smallest grouping, a double slash (//) for a deeper boundary of a higher-level grouping, and a triple slash (///) for the deepest boundary of the highest-level grouping. As in RPT, Extended RPT boundary markers are then summed across listeners, to provide an estimate of the perceived higher-level word groupings.

## Results

The analyses reported in this paper address three specific questions about the Stroke-Defined Gestures in the sample. First, is there a high proportion of Non-Referential gestures in this sample, as our preliminary impression suggested. Second, how do the strokes of these Non-Referential gestures align with the prosodic prominences of the speech they accompany. And third, do these Non-Referential gestures form a unified class. Before turning to these questions, we first summarize some of the characteristics of this sample.

### Corpus Characteristics

The 23-min portion of video in which the speaker was not occluded by graphics was labeled with 1,334 Stroke-Defined Gestures. The speech that accompanied these non-occluded regions was labeled with 2,065 Pitch Accent labels, 682 Full Intonational Phrase labels (ToBI Break Index 4), and 978 Intermediate Intonational Phrase labels (ToBI Break Index 3).

### Are Most of the Stroke-Defined-Gestures in This Corpus Non-referential?

Of the 1,334 SDGs identified in this sample, 1,263 (94.6%) were labeled as unambiguously Non-Referential), and 70 (5.4%) as Referential. (One gesture overlapped with a non-speech region and was omitted from further analysis.) This result confirms our initial informal impression that most of the manual co-speech gestures employed by this speaker are not referential. To our knowledge, extensive tabulations of the proportion of Referential vs. Non-Referential gestures are not available in the literature, so it is not yet possible to determine whether this proportion is atypically large. However, it appears that for this speaker, speaking in this style or circumstance, Non-Referential gestures predominate. This provides an opportunity to determine whether these largely Non-Referential co-speech gestures align with the prominent syllables of the speech, just as has been reported for individual Referential gestures and for corpora of gestures not sorted by their referentiality.

### Do These Gestural Strokes Align With Spoken Prominences?

In this study, alignment between a Stroke-Defined Gesture and a spoken prominence was defined as any degree of overlap between the temporal region labeled as an accented syllable and the region labeled as the gestural stroke. Although this definition of an association between strokes and accented syllables is more stringent than some in the literature, results are nevertheless consistent with earlier reports (Loehr, 2004, 2012; Renwick et al., 2004; Shattuck-Hufnagel et al., 2007), in that the proportion of strokes in gestures perceived as Non-Referential in this speech sample that overlap in time with accented syllables is very high: 83.1% (**Table 1**). This proportion does not differ substantially from that for the (much smaller number of) gestures perceived as Referential.

**Table 1:** The proportion of SDGs whose strokes overlap in time with a pitch-accented syllable, for gestures perceived as Referential vs. Non-Referential.

	Tokens with overlap of stroke w/PAcc syllable	Tokens with no overlap of stroke w/PAcc syllable	Total	Percent of tokens that overlap with a PAcc syllable
Referential	58	12	70	82.85%
Non-Referential	1,050	213	1,263	83.13%
Total	1,108	225	1,333	83.12%

This result suggests that, like the strokes of Referential gestures, the strokes of Non-Referential gestures tend to occur in conjunction with spoken prominences. That is perhaps unsurprising, in view of the general understanding of Non-Referential gestures as ‘beats’ which mark out the rhythm of the speech they accompany—but recall that these gestures were annotated from the video alone, without access to the accompanying speech. Thus this high percentage of overlap raises several interesting questions about how two types of alignment between spoken prominence and gestural stroke are related. On the one hand, the stroke of a Referential gesture is often aligned with a phrasally prominent syllable (see Kendon, 2004; Ch. 7), and on the other hand, the strokes of Non-Referential gestures in this sample are also reliably aligned with pitch-accented syllables. In discussions of the alignment of Referential-gesture strokes with phrasally prominent syllables, little mention is made of concepts such as ‘beating out the rhythm of the accompanying speech,’ whereas in discussion of the alignment of Non-Referential gesture strokes (or beats), this characterization is common. Future work will need to sort out whether the alignment phenomena for these two sets of co-speech gestures have a mechanism in common, or whether the alignment of Non-Referential gestures with the prominence patterns of the speech is, for example, more reliable in regions where the spoken prominences are more periodic, i.e., more beat-like.

### Do Groups of Gestures Align With Spoken Boundaries?

Our initial hypothesis about perceived gesture groupings was that they would align with intonational phrases, i.e., either with Intermediate Intonational Phrases (ToBI Break Index 3) or with Full Intonational Phrases (ToBI Break Index 4). However, analysis showed that this was not reliably the case. Only 224 of 431 PGGs (51.9%) fell within a single Full Intonational Phrase, so that many PGGs appeared to extend across more than one of these prosodic constituents. This result suggested that it would be useful to extend the analysis to higher-level constituents, which might be revealed by the Extended RPT labels. Results from this analysis will be presented in two sections, addressing (1) results from the E-RPT labeling suggesting that this method captures aspects of higher-level prosodic constituent structure, and (2) results from analysis of the gestures within such higher-level constituents, suggesting that gesture sequences within those constituents tend to share kinematics to a substantial degree.

#### Extended Rapid Prosodic Transcription

The expansion of Cole et al.’s (2010, 2014) method for rapid ‘crowd-sourced’ prosodic transcription to include marking three levels of perceived boundary was undertaken in an exploratory spirit, and the very preliminary results reported here must be taken as no more than suggestive. Nevertheless, they are thought-provoking, and so we include them here.

**FIGURE 3:**
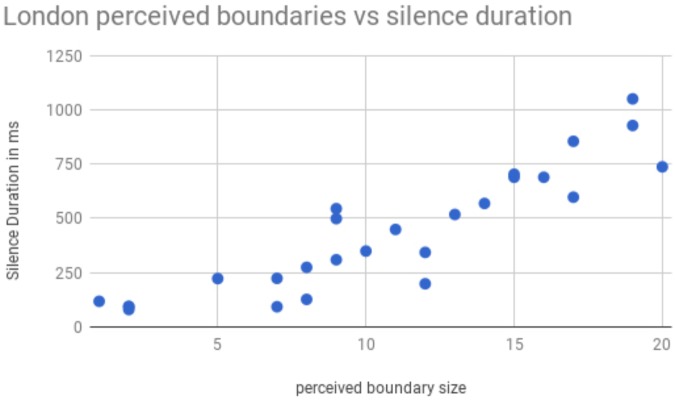
Perceived boundary size, as determined by the number of boundary markers inserted by listeners, shown as a function of the duration of silence between word pairs.

In this preliminary study, eight participants who were not experienced prosody labelers listened to the first 2 min 15 s of the London sample, and marked three levels of perceived boundary strength by inserting one, two or three forward slashes between pairs of words where they heard these boundaries. The number of slashes inserted by all eight participants was then totaled for each location where any participant inserted a boundary marker. Thus the highest number of boundary markers that was possible at any location was 3 × 8 or 24. **Figure 3** shows the total number of boundary markers inserted between a pair of words, as a function of an acoustic measure of the signal: the duration of the silence between those two words.

It appears that there is a reliable increase in the likelihood that the listener will perceive a boundary between two words, as a function of duration of the silence between those two words, so that the longer the silence, the more likely a listener is to insert a higher-level boundary. This observation is consistent with two inferences: that speakers organize their intonational phrases into higher level prosodic constituents, and that silence duration may be a reliable marker of these constituent boundaries. In an earlier study of possible groupings of intonational phrases into larger constituents, Wightman et al. (1992) also found hints of such a relationship between perceived higher-level constituent boundaries and silence duration. However, like the current findings, their study contained only a few such boundaries, so that the generality and reliability of the observation remains to be established by future studies.

#### Similarity of SDGs Within Higher-Level Constituents Identified on the Basis of E-RPT Judgments

**FIGURE 4:**
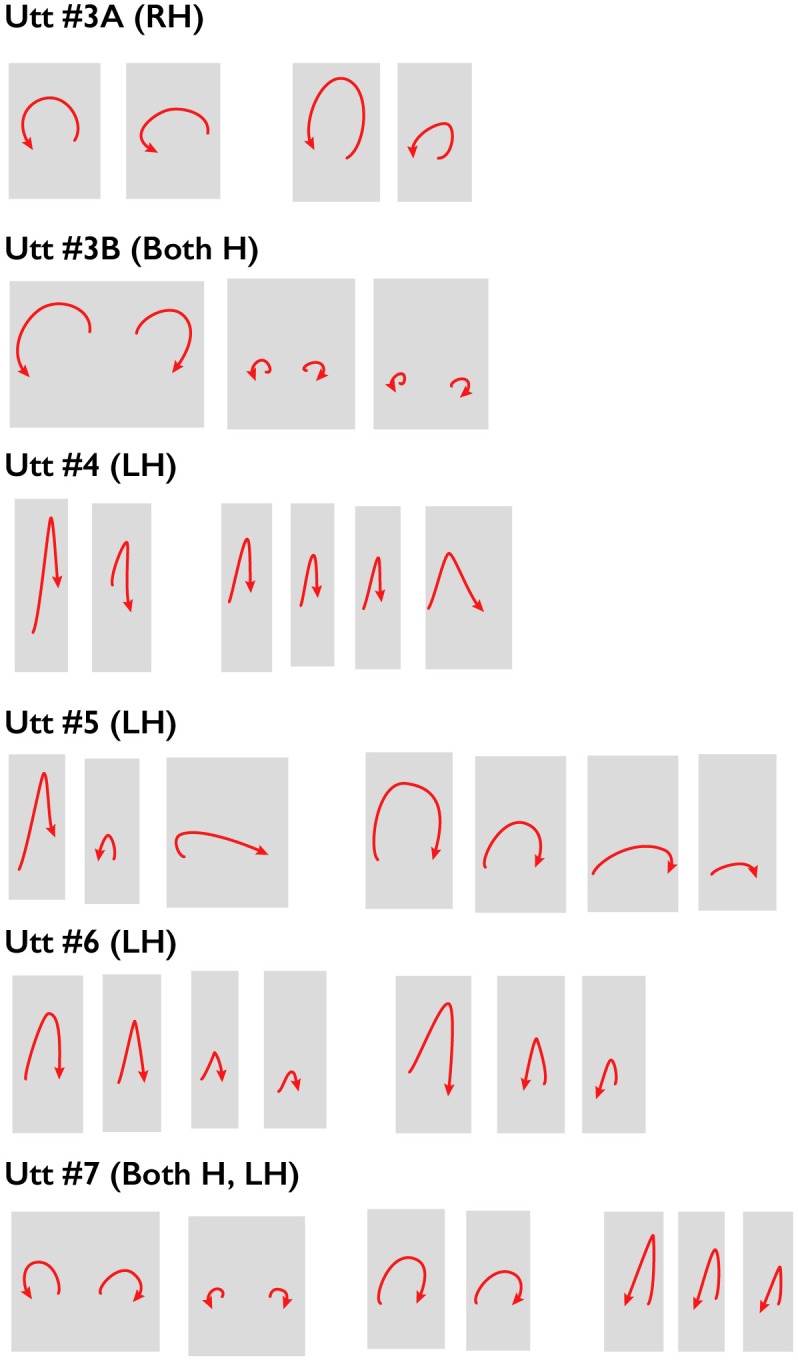
Gesture sketches for sequences of gestures that align with higher-level prosodic constituents (here called Utterances), as determined by Extended Rapid Prosodic Labelling, in the first 2 min 15 s of the London sample. Utt 1 contained no gestures, and during Utt 2 the speaker was mostly occluded by a graphic. Wider spaces indicate boundaries between successive Perceived Gesture Groups. See text for further explanation.

These preliminary results suggest that the listeners’ judgments reflect the silence-duration marker cue to higher-level constituents (other cues may of course also be contributing to the perception of these higher-level constituent boundaries), and they reflect a certain amount of agreement about the location of those constituents. On the assumption that this is the case, we adopted an arbitrary criterion of 15 or more boundary markers inserted by the annotators as an indicator of a higher-level grouping of individual utterances. This resulted in the identification of 8 higher-level constituents in this 2-min 15-s sample, compared to 66 Full Intonational Phrases and 100 Intermediate Intonational Phrases. Gestural sketches for the gestural accompaniments of 6 of the resulting 8 higher-level constituents are shown in **Figure 4**, where they are designated as Utterances. Visual inspection of these sketches suggests that, within a constituent defined in this way, the trajectory shape and handedness (right hand, left hand, or two hands) of successive gestures are quite similar, and that these characteristics differ from one such higher-level constituent to the next. Spacings between the sketches reflect somewhat smaller constituents defined by fewer than 15 E-RPT markings that group together smaller ToBI-labelled Full Intonational Phrases. Thus these preliminary data raise the possibility that closely related sequences of gestures are planned to occur within higher-level prosodic constituents.

### Do Non-referential Gestures Form a Unified Class?

The question of how to characterize Non-Referential co-speech gestures is an important one, because the convention of referring to them as ‘beats’ (or sometimes ‘batons’) makes it easy to assume that they form a homogeneous set. But a careful reading of the literature soon reveals that this is not the case. McNeill (1992), for example, distinguishes beats that are simple in-out or up-down ‘flicks’ of the hand or finger, and occur at specific points in a narrative, from other gestures that beat out the rhythm of the speech they accompany. Other researchers have also wrestled with the question of how to define and detect ‘beats,’ but from our point of view, a particularly interesting question concerns the ways in which a co-speech gesture can be seen as prosodic. That is, in what ways do gestures align with the prosodic prominences and constituents of the speech they accompany; in what ways to they have their own prominences and grouping structure; and in what ways do these two sets of prosodic behaviors align in time and in communicative function.

**FIGURE 5:**
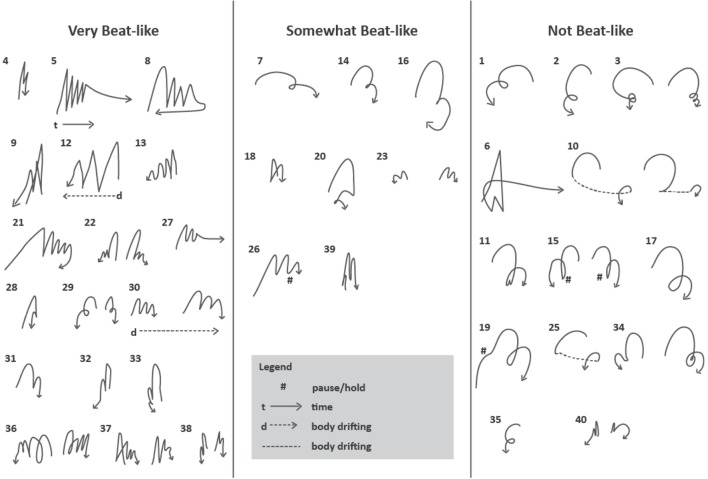
Gesture sequences judged to be beat-like, somewhat beat-like and not-beat-like for the first 43 PGGs in the London sample. For discussion of the characterization of these sets, see text.

In a preliminary attempt to address these questions, we developed a system for labeling the ‘beat-like-ness’ of a sequence of Stroke-Defined Gestures within a PGG. The definition of this characteristic was somewhat informal, and relates to whether the sequence of movements appears to be beating out a rhythm or not. Our first attempt used a simple binary decision: is this group of Stroke-Defined Gestures beat-like or not, but it soon became clear that a more nuanced system was needed. We settled on a three-level categorization: beat-like, somewhat beat-like and not beat-like. The middle category, somewhat beat-like, included sequences for which some of the strokes were perceived as beat-like and others were not. (The second author, who carried out this exploratory work, would like to try a 5-level system in the future.) Results of this annotation showed that 138 (32%) of the 431 PGGs contained gesture sequences that were perceived as beat-like. 119 were labeled as somewhat beat-like, and 174 as not beat-like. A gesture sketch summary for the first 41 PGGs in the London sample is shown **Figure 5**. It appears that gestures with a straight trajectory, performed in an up-and-down vertical dimension, are more likely to be perceived as beat-like, while those with a curved trajectory are less so. A second constraint appears to be temporal: strokes of gestures judged to be beat-like occurred in quicker succession than those judged not to be beat-like. For example, the mean inter-stroke interval, measured from the end of one stroke to the beginning of the next within a PGG, was 870 ms for sequences labeled as beat-like, 992 ms for sequences labeled as somewhat beat-like, and 1,119 ms for sequences labeled as not beat-like (excluding Perceived-Gesture-Group-final tokens, for which the interval to the end of the next stroke after the PGG boundary could be very long and variable).

This result provides an initial step in the direction of distinguishing the set of Non-Referential gestures that are perceived to have a strongly rhythmic beat-like character from those with different timing characteristics. Additional work will be needed to sort out the range of possibilities for characterizing different types of Non-Referential gestures, and the ways in which both Non-Referential and Referential gestures may have different timing relationships both with other gestures and with the speech they accompany.

This discussion highlights an additional issue of some importance, which is the question of whether the common practice of designating a co-speech gesture as a member of one or another mutually exclusive category, such as ‘beat-like’ or ‘iconic,’ could be usefully supplemented by a dimension-based system, in which each co-speech gesture is annotated for all of the characteristics that it exhibits. This would permit, for example, a sequence of iconic gestures to be labeled as beatlike, if it struck the viewer as beating out the rhythm of the speech. In his article for the Cambridge Encyclopedia of Linguistic Sciences, McNeill (2006) points out the advantages of such a dimension-based approach to co-speech gesture analysis:

“The essential clue that these are dimensions and not categories is that we often find iconicity, metaphoricity, deixis and other features mixing in the same gesture. Beats often combine with pointing, and many iconic gestures are also deictic…A practical result of dimensionalizing is improvement in gesture coding, because it is no longer necessary to make forced decisions to fit each gesture occurrence into a single box.”

Recently, Prieto et al. (2018) have discussed the multi-dimensional characteristics of beats in just these terms, and have proposed a labeling system that has many of these characteristics.

This approach may be particularly useful in the analysis of gestures which are not referential in any obvious way, but for which it is possible to imagine a metaphoric component. For example, if a speaker saying ‘And thus it came to pass…’ accompanies this spoken word sequence with a horizontal back-and- forth bimanual gesture with flat hands palm downward, as if smoothing a tablecloth, is that a metaphoric gesture that uses the indication of a flat smooth surface (or perhaps the act of smoothing) to stand for the concrete sequence of events to be described? This category of gesture is particularly interesting, because it encompasses gestures which bear an abstract relationship to the meaning of the speech. It sometimes seems as if almost any gestural movement can be thought of as having a metaphoric component, even though it is often difficult to put into words exactly what the potential metaphor is conveying. In a system where the degree of ‘metaphoricity’ could be ranked, or metaphoricity could be combined with other dimensions such as rhythmicity, such problems might be less vexing.

We note in passing that this sample includes very few of the hand or finger ‘flicks’ identified by McNeill (1992): only 23 examples of in-out flicks were identified, i.e., 1.7% of the total number of Stroke Defined Gestures. It is possible that this is due to the fact that this speaker was standing up behind a podium, with no place to rest his arms and hands, in contrast to speakers who produce a narrative while sitting in a chair with arms where they often rest their own arms and hands. This might make a finger-flick more comfortable. Another possibility is that in this sample, the function of a finger flick is served by a larger vertical movement of the entire arm and hand. However, that seems unlikely since such vertical movements are quite common in this sample, and often give the impression of being comprised of a preparation and a stroke, rather than of a bi-phasic in-out or up-down ‘flick.’

## Discussion

The observation that most of the co-speech gestures in this sample are judged to be Non-Referential has provided an opportunity to examine some of the characteristics of this type of gesture. The preliminary results presented here suggest that the strokes of these Non-Referential gestures align with prominent (i.e., pitch-accented) syllables in the speech they accompany, as has been reported for small samples of Referential gestures and for larger undifferentiated samples. In addition, preliminary observation raises the possibility that they group into constituents that align with higher-level prosodic constituents. This is consistent with the possibility of a parallel signaling of the organization of gestural and speech constituents at a level higher than the individual intonational phrase or even utterance, as proposed in, e.g., Kendon’s hierarchy of prosodic/gestural constituents (Kendon, 1972, 1980, 2004) and suggested by McNeill’s ‘cohesive’ gestures (McNeill, 1992). The observations reported here also suggest that Non-Referential co-speech gestures are not a homogeneous class, either kinematically or functionally, but instead may contain a wide variety of forms and serve a wide range of communicative ends. This raises the question of how the process of generating co-speech gestures can be integrated into current models of speech production planning. We will discuss each of these points in turn.

### The Alignment of Non-referential Gestures With Phrase-Level Prosodic Prominences

The question of how speakers determine the alignment of speech and co-speech gesture raises the methodological question of how best to define and study the alignment of spoken prosody with co-speech gestural events. With respect to the methodological question, a range of criteria for accent-gesture alignment have been used, from the strict temporal overlap of accented syllable with gestural stroke employed in this study, to a more expansive criterion of the two events being within a pre-defined number of milliseconds (e.g., Loehr, 2004), and from the alignment of temporal intervals (strokes with pitch-accented syllables) to the alignment of precise time points (F0 maxima, gesture apices). Brentari et al. (2013) suggest an interesting hierarchy of alignments, ranging from exact correspondence of the two time intervals, to overlap of the accented syllable with at least part of the stroke, to overlap with at least part of the entire gesture (including any preparation, hold, and recovery phases). They note that listeners can form an impression of which word a gesture is associated with, even when there is no direct temporal alignment. The question of what ‘counts’ as alignment/association between a spoken word and a gesture is clearly in need of investigation. Studies by Renwick et al. (2004), Yasinnik et al. (2004), and Shattuck-Hufnagel et al. (2007) measured alignment of manual strokes that had short sharp end points (resulting in a clear rather than a blurry video frame), which they called ‘hits,’ in a different set of academic lecture videos. Results showed that these end points occurred reliably toward the end of or just after a spoken accented syllable. Other investigations have focused on the alignment of the *onset* of a gesture or the *apex* of a stroke with an aspect of the speech. A model of speech production planning that includes gestural planning will need to specify which part of the gesture is planned to align with which part of the speech, in cases where that relationship is shown to be systematic.

Beyond the question of precisely how strokes and accented syllables are aligned, a larger question concerns which accented words and syllables are accompanied by co-speech gestures and which ones are not. For Referential gestures, earlier observations showed that the stroke is likely to overlap with a phrase-level prominence/pitch accent (e.g., Kendon, 2004). The finding that, in the sample of largely Non-Referential gestures examined here, 83% of the stroke intervals overlap in time at least partially with a pitch-accented syllable interval also reveals that 17% did not. Why are some strokes produced in non-accented regions of the speech? In addition, many pitch-accented syllables are not accompanied by a co-speech gesture. What determines which accents are aligned with strokes and which accents are not? This question awaits further study.

### The Alignment of Co-speech Gestures With Higher-Level Prosodic Constituents

The preliminary observation that perceived higher-level prosodic constituents in the speech may overlap with sequences of kinematically similar Non-Referential gestures raises the question of the precise nature of these constituents. Kendon (1980) notes that, in his observations, prosodic Tone Groups are combined into higher-level Locutions (said to generally comprise a complete sentence), which are in turn combined into Locution Clusters within a Discourse or conversational turn. These higher levels of constituent structure do not figure prominently in the Autosegmental-Metrical model of prosodic structure which was initially adopted for this study, in part because they have not been observed to have clear intonational markers. The Extended Rapid Prosodic Transcription method may prove useful in identifying acoustic cues that are specific markers for these higher level structures, like the duration-of-silence correlate discussed above. As Kendon proposed, some of the cues to these higher-order structures may be found in the gestural domain, in the sense that sequences of similar gestures may align with such constituents, so that a change in a gestural dimension might mark the start of a new constituent. If so, it will be consistent with the view that models of human speech production planning (and speech perception) must expand to accommodate the ways in which speakers insert this kind of information into the visual signal.

An interesting aspect of these preliminary observations is that they suggest subgroupings below the level of boundary corresponding to the arbitrary criterion adopted here (15 boundary markers). For example, in the set of gestures within the first of the higher-level spoken prosodic constituents shown in **Figure 4**, there appears to be a shift in gesture kinematics halfway through the constituent (i.e., between Utterance 3A and Utterance 3B); this corresponds to a location where the annotators inserted 14 boundary markers, a value which is just under our arbitrary threshold. The suggestion of a lower-level constituent boundary in the Extended Rapid Prosodic Transition data at that location is consistent with the change in gesture kinematics at that point. Similarly, in a later part of this sample, where listeners annotated the word sequence ‘and only nearly lost it, once’ as a single higher level constituent, but also indicated a smaller perceived boundary after ‘lost it,’ the trajectory shape of the Stroke-Defined Gesture produced with ‘once’ is different from that of the SDGs produced with the preceding word sequence ‘and only nearly lost it.’ Such observations support the possibility that, like prosodic constituents, gesture sequences are hierarchically organized.

Finally, the question of what signals the grouping of a sequence of gestures into a constituent has been only tangentially addressed in this paper. In the larger ongoing project of which this study is a part, the visual-only annotation of gesture groups employed to identify PGGs is supplemented with gesture phase labeling. This will enable testing the hypothesis advanced in Kendon (2004) that groups of gestures (Gesture Units, in his terminology) are marked by a recovery phase at the end of the group-final gesture, in this sample of academic-lecture-style speech that contains mostly Non-Referential gestures. Moreover, combining video-only labeling of PGGs (which focuses on the physical characteristics and timing of the gestural movements), with sound-only labeling of the spoken prosodic constituents, allows the investigation of timing and grouping alignments between the two streams of behavior. In the end, however, by combining these separate annotation approaches with listening and looking at the same time, it may be possible to determine how the semantic, syntactic, prosodic, and gestural structures of an utterance combine to form an effective act of communication. Modeling that process will require a collaborative effort which, it is hoped, this report may help to inspire.

### Integrating Gesture Production Planning Into Current Speech Production Models

The results described in this paper offer support for the hypothesis put forward over the years by Kendon and McNeill and others, that the gestures that accompany a spoken utterance are an integral part of the communication signal, and thus that the planning process for producing a spoken utterance must include the planning of co-speech gestures. In particular, taken together with other results in the literature, these findings suggest a tight temporal coordination between the prosodic structure (i.e., the grouping and prominence structure) of a spoken utterance and the prosodic structure of the gestures that accompany it. In this way they are also consistent with the hypothesis proposed by Keating and Shattuck-Hufnagel (2002), that the planning frame for a spoken utterance is a prosodic planning frame. Keating and Shattuck-Hufnagel propose a ‘Prosody First’ model of the phonological encoding process in speech production planning. In that model, a representation of the phrase-level prosody of an utterance is computed as an abstract structure, simple at the beginning of the planning process but gaining complexity as the phonological elements of the planned utterance are inserted into its sequentially and hierarchically organized slots. On this view, the prosodic structure of an utterance provides the representational ‘spine’ that governs the serial ordering of lexical elements and their sub-constituents, the integration of multiple factors involving the surface timing/duration patterns of the speech signal, and the computation of surface timing patterns.

For a more comprehensive view of speech production planning that begins with the earliest formation of the intended message, one can turn to the model proposed by Levelt (1989) and implemented by Levelt et al. (1999). In this model the initial formulation of a message takes place in terms of a cognitive representation of meaning that is pre-linguistic. It may be this very early representation that guides the subsequent formation of both the spoken and the gestural realizations of the utterance. Kendon (1980) suggests this when he notes that

“we may mention the views of Chafe (1970) who has argued explicitly for the position that the process of utterance generation proceeds through a series of steps starting with the organization of semantic structures. The work on gesticulation we have reviewed here would suggest that this earliest stage in the process of utterance formation has, or can have, direct expression in gesticular action.” (p. 224)

Jannedy and Mendoza-Denton (2005) expressed a related idea in their report of a study of co-speech gesture timing and function in a sample of highly emotional (and presumably not highly rehearsed) political speech:

“We found that speech and gesturing are two different channels/modes of information transfer which allow for different content to be transmitted. If we assume the validity of Bolinger’s (1986) claim that “gesture and speech stem from the same semantic intent […],” then we commit ourselves to the notion that some degree of pre-planning is involved in generating not only speech output but also gestural output in order to convey information on different planes. How information is structured and divided up across the two channels is not understood at this point. From our data it appears that complementary and contextual information is transmitted via gestures while concrete assertions are made explicit via speech. We also do not know what constraints exist in (pre-)planning complex gestures that we know are time-aligned with linguistic structure in the final output.” (Jannedy and Mendoza-Denton, 2005, p. 233).

## Conclusion

The results described in this paper offer support for the hypothesis put forward over the years by Kendon and McNeill and their colleagues, that the gestures that accompany a spoken utterance are an integral part of the communication signal, and thus that the planning process for producing a spoken utterance must include the planning of co-speech gestures. In particular, taken together with other results in the literature, these findings suggest a tight temporal coordination between the prosodic structure of a spoken utterance and the prosodic structure of the gestures that accompany it. In this way they are also consistent with the hypothesis proposed by Keating and Shattuck-Hufnagel (2002), that the planning frame for a spoken utterance is a prosodic planning frame. On this view, the prosodic structure of an utterance provides the representational ‘spine’ that governs not only the serial ordering of lexical elements and their sub-constituents, the integration of multiple factors involving the surface timing/duration patterns of the signal, and the computation of surface timing patterns, but also, potentially, the integration of auditory with visual aspects of the speech act. On this hypothesis, the prosodic planning frame governs the timing of occurrence and the duration of various components of both the spoken and the gestural aspects of the communicative act (Shattuck-Hufnagel et al., 2016).

It must be emphasized that the results reported here are drawn from a single speaker, producing speech in a particular context and style, and it is not yet clear how far they will generalize. Moreover, although some of the results reported here are based on large numbers of manual gestures and spoken prosodic events, others are based on very small numbers of observations and are thus highly preliminary. However, in concert with other observations in the literature, the results reported here open the door to a number of lines of study. These include the investigation of the cues to higher-level prosodic constituents that group spoken intonational phrases together, and of the patterns in the use of individual cues to prosodic constituents in both the spoken and the gestural domains that may vary across speakers, listeners, learners, and users of different languages. It appears that the study of how co-speech gestures and speech interact in communication systems is poised on the threshold of some very interesting discoveries which will enlarge and enhance our ability to build models of the speech production planning and speech perception processes.

## Ethics Statement

This work was carried out under approval of MIT’s Committee on the Use of Human Experimental Subjects (COUHES).

## Author Contributions

SS-H generated the hypotheses to be tested, developed the Extended RPT method, labeled the prosody, participated in the development of the gesture labeling methods and analysis of the data, and wrote most of the text. AR labeled the gestures, developed the gesture sketch method, participated in the development of the gesture labeling methods and data analysis, and generated the figures.

## Conflict of Interest Statement

The authors declare that the research was conducted in the absence of any commercial or financial relationships that could be construed as a potential conflict of interest.

## Footnotes

1 https://www.thegreatcourses.com/

2 https://tla.mpi.nl/tools/tla-tools/elan/

3 http://www.fon.hum.uva.nl/praat/

4 https://ocw.mit.edu/courses/electrical-engineering-and-computer-science/6-911-transcribing-prosodic-structure-of-spoken-utterances-with-tobi-january-iap-2006/
